# Hypocapnia Alone Fails to Provoke Important Electrocardiogram Changes in Coronary Artery Diseased Patients

**DOI:** 10.3389/fphys.2019.01515

**Published:** 2020-01-20

**Authors:** Michael J. Parkes, James P. Sheppard, Thomas Barker, Aaron M. Ranasinghe, Eshan Senanayake, Thomas H. Clutton-Brock, Michael P. Frenneaux

**Affiliations:** ^1^School of Sport, Exercise and Rehabilitation Sciences, University of Birmingham, Birmingham, United Kingdom; ^2^National Institute for Health Research/Wellcome Trust Birmingham Clinical Research Facility, University Hospitals Birmingham NHS Foundation Trust, Birmingham, United Kingdom; ^3^Department of Cardiovascular Medicine, University of Birmingham, Birmingham, United Kingdom; ^4^Department of Anaesthesia and Intensive Care Medicine, University of Birmingham, Birmingham, United Kingdom

**Keywords:** hypocapnia, coronary artery vasoconstriction, electrocardiogram, angina, mechanical hyperventilation, calcium

## Abstract

**Background:**

There is still an urgent clinical need to develop non-invasive diagnostic tests for early ischemic heart disease because, once angina occurs, it is too late. Hypocapnia has long been known to cause coronary artery vasoconstriction. Some new cardiology tests are accompanied by the claim that they must have potential diagnostic value if hypocapnia enhances their cardiac effects in healthy subjects. But no previous study has tested whether hypocapnia produces bigger cardiac effects in patients with angina than in healthy subjects.

**Methods:**

Severe hypocapnia (a PetCO_2_ level of 20 mmHg) lasting >15 min was mechanically induced by facemask, while conscious and unmedicated, in 18 healthy subjects and in 10 patients with angina and angiographically confirmed coronary artery disease, awaiting by-pass surgery. Each participant was their own control in normocapnia (where CO_2_ was added to the inspirate) and the order of normocapnia and hypocapnia was randomized. Twelve lead electrocardiograms (ECG) were recorded and automated measurements were made on all ECG waveforms averaged over >120 beats. 2D echocardiography was also performed on healthy subjects.

**Results:**

In the 18 healthy subjects, we confirm that severe hypocapnia (a mean PetCO_2_ of 20 ± 0 mmHg, *P* < 0.0001) consistently increased the mean T wave amplitude in leads V1–V3, but by only 31% (*P* < 0.01), 15% (*P* < 0.001) and 11% (*P* < 0.05), respectively. Hypocapnia produced no other significant effects (*p* > 0.05) on their electro- or echocardiogram. All 10 angina patients tolerated the mechanical hyperventilation well, with minimal discomfort. Hypocpania caused a similar increase in V1 (by 39%, *P* < 0.05 vs. baseline, but *P* > 0.05 vs. healthy controls) and did not induce angina. Its effects were no greater in patients who did not take β-blockers, or did not take organic nitrates, or had the worst Canadian Cardiovascular Society scores.

**Conclusion:**

Non-invasive mechanical hyperventilation while awake and unmedicated is safe and acceptable, even to patients with angina. Using it to produce severe and prolonged hypocapnia alone does produce significant ECG changes in angina patients. But its potential diagnostic value for identifying patients with coronary stenosis requires further evaluation.

## Introduction

In patients with angiographically confirmed coronary artery disease, voluntary hyperventilation-induced hypocapnia to ∼20 mmHg causes some coronary artery constriction (Rowe et al., 1962; Neill and Hattenhauer, 1975). In healthy subjects, voluntary hyperventilation is also variously reported to cause ECG changes (Rutherford et al., 2005) and to enhance the effects of non-invasive techniques that may assess myocardial oxygenation (Guensch et al., 2013). Furthermore there is a greater fall in their myocardial OS-CMR signal (−11%) with voluntary hyperventilation than the rise in this signal (+4%) with adenosine (Fischer et al., 2014). Assessing coronary vascular function using voluntary hyperventilation-induced hypocapnia as an endogenous vasoconstrictor is also suggested as a clinically exciting prospect because it is safe, non-invasive and presumably free of the side effects of pharmacological effects in combination with contrast agents (Guensch et al., 2013; Fischer et al., 2014). But the benchmark is whether hypocapnia produces bigger effects in patients with myocardial ischemia. To our knowledge this has not previously been studied (Rutherford et al., 2005; Fischer et al., 2014; Crystal, 2015).

Voluntary hyperventilation is difficult for both healthy subjects and patients and produces short periods (a few minutes) of unstable hypocapnia (Rutherford et al., 2005). Non-invasive mechanical hyperventilation by facemask is a superior technique for inducing long and stable periods of severe hypocapnia. We have already established that this is easy for conscious, unmedicated and healthy subjects, and for patients with breast cancer (Parkes et al., 2016b), and produces long periods (up to 1 hr) of stable hypocapnia as severe as can safely be applied to patients (Cooper et al., 2003, 2004; Rutherford et al., 2005; Sheppard et al., 2011; Parkes et al., 2019). Since mechanically induced hypocapnia is so simple, cheap and comfortable (for patients) to perform, it is potentially viable as a diagnostic modality for identifying patients with fixed coronary stenoses. Furthermore, non-invasive mechanical ventilation is now being considered to assist radiotherapy, both for cardiac ablation and for radiotherapy delivery to reduce respiratory motion of thoracic and abdominal cancers (Parkes et al., 2016b; West et al., 2018; Van Ooteghem et al., 2019). There is therefore a need to demonstrate whether it would be well tolerated in patients with co-morbid coronary artery disease.

Here we test for the first time whether angina patients can be mechanically hyperventilated non-invasively. This technique can induce more severe and prolonged hypocapnia than can be induced by voluntary hyperventilation. We therefore tested whether this hypocapnia induces ECG and echocardiographic changes in healthy controls and in patients with angina due to demonstrated coronary artery disease. In this small proof of concept study, we also evaluated whether these changes were greater in patients with coronary artery disease than in healthy controls.

## Materials and Methods

Experiments were conducted in the NIHR/WTCRF at the University Hospital Birmingham. All participants gave informed consent and all experiments were approved by the Walsall Local Research Ethics Committee and were performed in accordance with the Declaration of Helsinki and Good Clinical Practice. As an additional safety precaution, experiments on angina patients were conducted in the presence of a currently trained intensive care unit nurse.

We tested 18 normal healthy subjects (aged 20–30 years, 13 males) with no known cardiovascular disease and who were not smokers. We also tested 10 male patients aged 43–72 years with history of typical angina pectoris and angiographically confirmed severe coronary heart disease who were awaiting bypass surgery. Patients had stable exertional angina without rest pain or ECG abnormalities. 5/10 had triple vessel disease and all clinical details and medications are given in Table 1. Patients taking prophylactic long acting nitrates had these discontinued 36 h in advance and none had taken short acting nitrates for 2 h prior to testing.

**TABLE 1:** Patient details and medication.

Patient	Age	BMI	Hyper-tension	Diabetic	Ventricular function	Low dose aspirin	NYAA	CCS	Iso-sorbide	βblocker	Nicorandil	ACE inhibitor
1	54	26	No	No	Good	Yes	1	2		Yes		
2	72	26	Yes	No	Good	Yes	2	2	Yes	Yes		Yes
3	43	22	No	No	Good	Yes	n/a	n/a	Yes			
4	75	31	No	No	Half	Yes	2	2			Yes	
5	65	32	Yes	No	Good		2	2	Yes	Yes	Yes	
6	68	23	Yes	No	Good	Yes	2	2	Yes	Yes		Yes
7	57	28	Yes	Yes	Good	Yes	2	3	Yes	Yes	Yes	Yes
8	52	31	No	No	Good	Yes	2	3	Yes	Yes		
9	64	32	Yes	No	Good	Yes	1	3	Yes	Yes		
10	57	36	yes	Yes	Good	Yes	2	3		Yes		Yes

			**Vessel disease >50% stenosis with corresponding leads where ECG changes might be expected**									

**Patient**	**Stent**		**Previous MI.**		**R/L dominant**	**Vessel**	**V1-V4**		**V4-V6**			**aVF, II, and III**

1	Yes		Yes		Right	Triple	Prox LAD		Circumflex			R coronary block
2					Right	Triple	Prox LAD		Prox circumflex			R coronary
3					Right	Single	Prox LAD		–			–
4					Right	Double	LAD block		Prox circumflex			–
5					Right	Double	Prox LAD		Circumflex (OMI)			–
6					Left	Triple	Prox LAD		Circumflex			–
7					Left	Triple	LAD block		Prox circumflex (distal blocked)			–
8					Right	Triple	Prox LAD		Circumflex			R coronary
9					Left	Single	Prox LAD		–			–
10	Yes		Yes		n/a	Double	Prox LAD		Prox circumflex			(Inferior MI.)

*n/a, not available.*

Blood pressure was recorded continuously using a finger plethysmograph (Finapres 2300) and end tidal partial pressure of carbon dioxide (PetCO_2_) via an in-line capnograph (Hewlett Packard 78354A). All data were recorded by a CED1401 (Cambridge Electronic Design). Antecubital venous catheters were inserted in 14 of the healthy subjects. Because hypocapnia is often believed to cause electrolyte changes (Rutherford et al., 2005), one 10 ml sample was taken at the end of the normocapnia and hypocapnia periods and ionized Ca^2+^ and K^+^ levels were measured using a blood gas analyzer (Rapid Lab 865).

In the 18 healthy subjects, echocardiographic recordings were also performed as an independent measure of any potentially ischemic changes in heart function during hypocapnia. 2D echocardiography was used to assess wall motion, Doppler flow analysis was used to measure diastolic function and tissue Doppler analysis was used to measure myocardial wall velocity.

All participants were conscious and unsedated, without previous experience of non-invasive mechanical hyperventilation by facemask. All were positioned semi-recumbent. Mechanical hyperventilation by facemask in air was performed (Cooper et al., 2003, 2004; Rutherford et al., 2005; Parkes et al., 2014) at 16 breaths per minute and with an inflation volume increased to induce a PetCO_2_ level of 20 mmHg. All had 1–2 practice sessions in which all experimental maneuvers were performed on previous days. Each participant was their own control and, for the control (normocapnia) condition, we applied the same inflation frequency and inflation volume but added sufficient flow of 5% CO_2_ in air to restore their PetCO_2_ to their own eupneic levels. All had two sessions of mechanical hyperventilation on separate days where normocapnia and hypocapnia were established for at least 15 min, and applied in random order, before making the 2-min recordings. Therefore, only if changes were consistently reproducible in all experiments, over multiple subjects and repeatable on two separate days would they be statistically significant.

Twelve lead ECG waveforms were calculated using standard formulae (Wagner, 2008; Sheppard et al., 2011). Signal averaging techniques (Rutherford et al., 2005) were used to maximize sensitivity in detecting any consistently reproducible ECG changes, with averaging performed “blind” using fully automated algorithms for 2 min periods (∼120 beats) to derive ECG amplitude in mV [P, Q, R, ST segment (S, J slope, J, J80 height), RS and T heights] and timing in seconds (QT, QTc, PR, and QRS intervals, Q and T duration). We then calculated the mean measurement for the two normocapnia and hypocapnia sessions for each subject. Measurements were also validated (Sheppard et al., 2011) with an electronic ECG simulator (Dynatech Nevada Inc., Model 212B, Nevada) and against a Philips Hewlett Packard Pagewriter 200. Our analysis has the sensitivity to detect ECG changes two orders of magnitude lower that the clinical thresholds of 0.1 mV.

Since ECG amplitude decreases as inflation volume increases (Rutherford et al., 2005), clinical thresholds depend on the inflation volume used. To adjust these thresholds for the hyperventilation inflation volume we used, in each lead we measured the percentage change in T wave amplitude in healthy subjects between eupnea and the volume at which each was hyperventilated and adjusted each clinical threshold accordingly (see Table 2). Recent guidelines also recommend consideration of gender differences in the ventricular repolarization phase of the cardiac cycle (Surawicz and Parikka, 2002) for leads exhibiting the largest T wave and ST segments i.e., transverse plane leads (Wagner et al., 2009). So we also took gender into account.

**TABLE 2:** clinical threshold changes for eupnea and hyperventilation.

**Lead**	**AHA clinical threshold**	**AHA clinical threshold change**
	**change in eupnea**	**adjusted for hyperventilation**
I	>1.0 mV	>0.4 mV
II	>1.0 mV	>0.5 mV
III	>1.0 mV	>0.8 mV
aVR	>1.0 mV	>0.4 mV
aVL	>1.0 mV	>0.3 mV
aVF	>1.0 mV	>0.5 mV
V1	>1.0 mV	>2.1 mV
V2	>1.0 mV	>1.0 mV
V3	>1.0 mV	>1.0 mV
V4	>1.0 mV	>0.9 mV
V5	>1.0 mV	>1.2 mV
V6	>1.0 mV	>0.9 mV

Table 2 shows the absolute thresholds (mV) and our adjusted thresholds indicating clinical significance for the ECG measured in eupnea from the American Heart Association (AHA), American College of Cardiology Foundation (ACCF) and Heart Rhythm Society (HRS) (Wagner, 2008; Surawicz et al., 2009; Wagner et al., 2009).

Statistical analysis used a two tailed paired *t*-test with *p* < 0.05 taken as significant, with each participant’s measurements in normocapnia paired with their own measurements in hypocapnia. We have not applied the Bonferroni correction for ECG statistical analysis because we cannot assume all 12 ECG leads are independent and uncorrelated, so this correction is too conservative. Comparison between healthy subjects and patients used an unpaired *t*-test. All data presented is expressed as mean ±standard error. Statistical review of this study was performed by a biomedical statistician.

### Supportive Funding

This work was supported, and presents independent research commissioned, by the National Institute for Health Research (United Kingdom) under the National Program on New and Emerging Applications of Technology Grant (FSD007).

### Role of the Funding Source

The funding sources had no role or involvement in the writing of the manuscript or the decision to submit it for publication. Examples of involvement include: data collection, analysis, or interpretation; trial design; patient recruitment; or any aspect pertinent to the study.

## Results

### Hypocapnia in 18 Healthy Subjects

It took only a few minutes to accustom healthy subjects to mechanical hyperventilation. This caused significant decreases in PetCO_2_ (falling by 18 mmHg, from 38 ± 0 mmHg to 20 ± 0 mmHg, *P* < 0.0001), in mean arterial pressure (by 9 ± 3 mmHg, from 94 ± 4 mmHg, *P* < 0.01) and a trivial decrease in calcium levels (by only 0.03 ± 0.01 mmol/l from 1.2 ± 0.0 mmol/l, *P* < 0.001). It significantly increased mean heart rate (by 4 ± 1 b.p.m. from 58 ± 2 b.p.m., *P* < 0.01) and had no significant effects on plasma potassium levels (4.0 ± 0.1 mmol/l).

**FIGURE 1:**
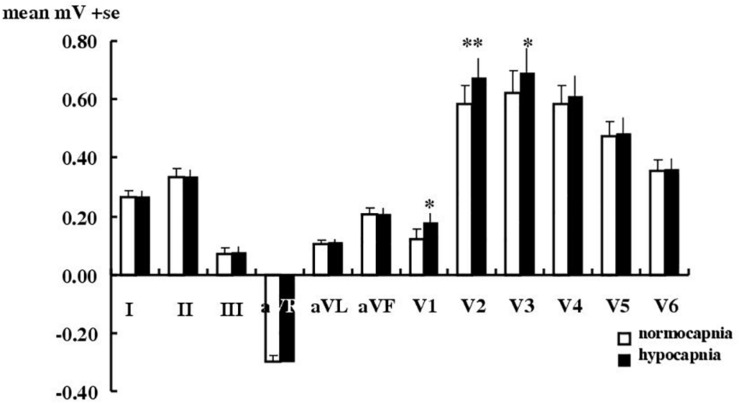
Effect of hypocapnia on the T wave in healthy subjects. Mean ± SE T wave amplitude in 18 healthy subjects during normocapnia or hypocapnia. ^∗^*P* < 0.05, ^∗∗^*p* < 0.01, paired *t*-test.

Figure 1 shows that for limb leads, hypocapnia caused no significant T wave changes. For chest leads it caused a significant change only in leads V_1_–V_3_ (mean elevations, respectively of 31% i.e., 0.06 ± 0.01 mV, *P* < 0.01; of 15% i.e., 0.09 ± 0.02 mV, *P* < 0.001; and of 11%, i.e., 0.07 ± 0.02 mV, *P* < 0.05). All changes remained within clinically acceptable limits for normal T wave amplitude changes (Table 2). Significant T wave elevation occurred in males in leads V_1__–__3_, but only in V_1_ in females. T wave elevation during hypocapnia was not accompanied by consistent changes of any of the 12 ECG leads in the ST segment nor other amplitudes (P, Q, R, RS heights) or timings (QT, QTc, PR, QRS intervals, Q or T durations).

Echocardiography was feasible and straightforward during mechanical hyperventilation. But in our healthy subjects it was even less sensitive than electrocardiography in that it failed to detect any significant effects of hypocapnia on wall motion or diastolic function i.e., no significant effects nor changes beyond clinically normal values on wall motion [LVID difference, −1.80 ± 0.0 cm [normocapnia] vs. −1.81 ± 0.0 cm (hypocapnia), *P* > 0.90; fractional shortening, 36 ± 1% (normocapnia) vs. 34 ± 2% (hypocapnia), *P* > 0.10], diastolic function [E/A ratio, 1.99 ± 0.13 [normocapnia] vs. 1.78 ± 0.14 (hypocapnia), *P* > 0.10; deceleration time 0.20 ± 0.01 s (normocapnia) vs. 0.21 ± 0.01 s (hypocapnia), *P* > 0.45] nor on diastolic wall velocities in both the septum [12.6 ± 0.5 cm/s (normocapnia) vs. 12.4 ± 0.3 cm/s (hypocapnia), *P* > 0.60] and lateral wall [16.7 ± 0.6 cm/s (normocapnia) vs. 16.4 ± 0.5 cm/s (hypocapnia), *P* > 0.60]. For this reason, echocardiography was not pursued in our angina patients.

### Hypocapnia in 10 Angina Patients

Mechanical hyperventilation (lasting up to ∼1 h) and hypocapnia were as safe and easy to accustom and apply in angina patients as in healthy subjects. During mechanical hyperventilation, none could distinguish between normocapnia and hypocapnia (Cooper et al., 2004; Rutherford et al., 2005) and none reported any distress or particular discomfort. More importantly, none experienced angina. Mechanical hyperventilation caused mean PetCO_2_ levels to decrease (from 38 ± 0 mmHg to 20 ± 0 mmHg, *P* < 0.0001) with no significant effects on mean heart rate (61 ± 9 b.p.m.) nor in mean arterial pressure (92 ± 6 mmHg). Again, none experienced angina.

Based on the location of the site of their coronary stenoses, we expected hypocapnia induced coronary artery constriction to produce more pronounced T wave changes for all 10 patients in V1–V4, for 8/10 patients in V4–V6 and for 4/10 in II, III, and aVF. No such pronounced changes were found.

**FIGURE 2:**
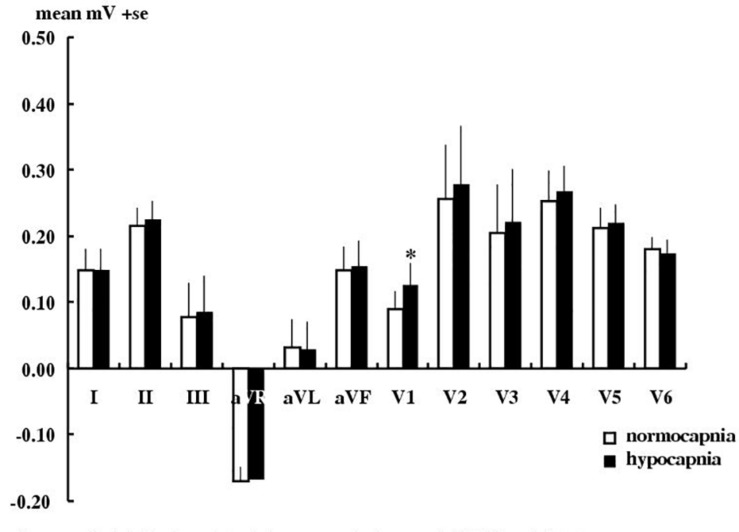
Effect of hypocapnia on the T wave in angina patients. Mean ± SE T wave amplitude in 10 angina patients during normocapnia or hypocapnia. ^∗^*P* < 0.05, paired *t*-test.

Figure 2 shows that hypocapnia did cause significant ECG changes in angina patients, but these changes were not significantly greater than those observed in healthy subjects (*P* > 0.05 by unpaired *t*-test) and ST depression (the usual hallmark of subendocardial ischemia) was not observed. Thus hypocapnia caused significant changes in only lead V1 T wave (a 39% elevation, by 0.035 ± 0.014 mV, *p* < 0.05), but no other measurable nor consistent ECG change in either amplitude (P, Q, R, RS, ST segment heights) nor in timing (QT l, QTc, PR, QRS intervals, Q, T durations).

Performing the same analysis above on only the 5 patients with triple vessel disease showed no greater or more consistent effects of hypocapnia on ECG amplitude or timing. Similarly, inspection of individual results revealed no obviously greater effects in patients who never took β blockers (#3, #4) or never took organic nitrates (#1, #4, #10) or in those with the worst Canadian Cardiovascular Society scores (#7, #8, #9, #10).

## Discussion

Since the 1930s, clinical and often anecdotal reports have suggested that voluntary hyperventilation-induced hypocapnia might affect the electrocardiogram (McCance, 1932). Surprisingly ours is the first systematic test of whether hypocapnia produces effects in patients with angina that are any greater than those observed in healthy subjects. Despite our producing more consistent and severe hypocapnia, in more patients, in more consistently ill patients (all with angiographically confirmed coronary heart disease, exertional angina and awaiting bypass surgery) and for longer durations than any previous studies (McCance, 1932; Rowe et al., 1962; Neill and Hattenhauer, 1975; Freeman and Nixon, 1985; Kruyswijk et al., 1986; Rutherford et al., 2005; Guensch et al., 2013; Fischer et al., 2014), we found that ECG changes were no more marked in patients with angina than in healthy controls.

The most severe hypocapnia that can safely be achieved in humans during hyperventilation with air (without causing tetany) is an arterial partial pressure of carbon dioxide (PaCO_2_) of 20 mmHg (Rutherford et al., 2005). This causes severe constriction of epicardial resistance vessels (Rowe et al., 1962; Neill and Hattenhauer, 1975) and reduces coronary blood flow by up to 30% (Rowe et al., 1962; Neill and Hattenhauer, 1975; Yokoyama et al., 2008; Crystal, 2015). The reduced coronary blood flow, possibly coupled with the increased the affinity of hemoglobin for O_2_ (Neill and Hattenhauer, 1975), is thought to have the capability of reducing oxygen supply to the myocardium (Guensch et al., 2013; Fischer et al., 2014; Crystal, 2015). This is despite the fact that classic respiratory physiology (according to the alveolar gas equation) demonstrates that lowering PaCO_2_ by 20 mmHg must also raise alveolar PO_2_ by and equal 20 mmHg. Such voluntarily-induced hypocapnia does provoke angina very occasionally in some patients with stable coronary artery disease (Rowe et al., 1962; Neill and Hattenhauer, 1975; Crystal, 2015). It also provokes a characteristic focal epicardial coronary artery spasm with coronary occlusion in the very small number of patients suffering from Prinzmetal’s angina (Pepine et al., 1992) and has been used as a diagnostic test for Prinzmetal’s angina.

But to develop a diagnostic test that consistently reveals in every patient with early coronary artery disease any haemodynamically significant coronary plaque, and or provokes coronary artery spasm, the key question is whether hypocapnia produces greater effects in patients with established ischemic heart disease than in healthy volunteers. We undertook our study since the potential diagnostic utility of hyperventilation induced hypocapnia to identify patients with fixed coronary artery stenosis has not been investigated previously. This is despite almost every new non-invasive test being claimed as potentially important if hypocapnia enhances its results in healthy subjects.

Even in healthy subjects, it has never been satisfactorily established whether hypocapnia does consistently affect the ECG, because previously voluntary hyperventilation was always used. The reliability and reproducibility of voluntary hyperventilation is questionable (Rutherford et al., 2005). This is because of its limitations in inducing sufficiently stable, severe, and reproducible hypocapnia and the additional pressor changes and ECG movement artifacts from the large variations in voluntary chest inflation. Thus it only caused ischemic changes in the T wave, ST segment and QTc interval in 8–57% of healthy subjects (McCance, 1932; Christensen, 1946; Wasserburger et al., 1956; Golden et al., 1975; Joy and Trump, 1981) and does not even always affect angina patients (Kenigsberg et al., 2007).

We previously developed a method (Rutherford et al., 2005) in healthy volunteers to induce prolonged hypocapnia by using mechanical hyperventilation with a facemask. This is easily tolerated in conscious and unmedicated subjects and provides an entirely uniform pattern of lung inflation and more severe, prolonged and stable hypocapnia at 20 mmHg without any consistent pressor or electrolyte changes. Indeed such non-invasive mechanical hyperventilation has widespread clinical application e.g., for radiotherapy to assist cardiac ablation and to reduce movement during all radiotherapy in the thorax and abdomen (Parkes et al., 2016a, b; West et al., 2018; Van Ooteghem et al., 2019). But there is still little clinical awareness of the simplicity, availability, and safety of this technique. We also developed a method to standardize ECG waveform measurement, by averaging it over ∼120 heart beats (Rutherford et al., 2005), so that only consistently reproducible effects would be found. Whereas previous ECG analysis techniques tended to select only the occasional and discrepant ECG waveforms. With our techniques we found that hypocapnia significantly decreased T wave amplitude in a modified lead I in 13/15 healthy subjects by a mean of 0.1 mV (Rutherford et al., 2005). Furthermore, we recently validated a highly sensitive and fully automated averaging system for 12 lead ECG measurement to perform this analysis on patients and specified precisely what filtering and other waveform manipulation was applied (Sheppard et al., 2011). Our new automated system was applied here.

### Hypocapnia in Healthy Subjects

Previously for healthy subjects (Rutherford et al., 2005) we used a modified three lead ECG using an analog amplifier and analyzed data manually. Here we used digital amplifiers and our “blind”, more sophisticated and rigorous calibration and analysis (Sheppard et al., 2011), with changes compared to the latest clinical thresholds (Table 2) set out by the AHA, ACCF, HRS (Wagner, 2008; Rautaharju et al., 2009; Surawicz et al., 2009; Wagner et al., 2009). Here the incidence of T wave changes with hypocapnia (in 61% of subjects in lead I) is less than the 87% we found previously in our group of different healthy subjects (Rutherford et al., 2005) and the amplitude change is smaller too. Nevertheless we confirm here that mechanically induced hypocapnia in a new set of healthy subjects still produces consistent T wave effects (increases of up to 31%, 0.06–0.09 mV in leads V_1__–__3_). It is possible that these ECG changes do reflect some vasoconstriction caused by hypocapnia. These ECG effects, however, are small, are below the current AHA clinical thresholds and were not accompanied by any significant changes in any other features of the 12 lead ECG. Neither could we detect hypocapnia causing any impairment in cardiac function using echocardiography in healthy subjects.

This small effect on the T wave is not caused by changes in plasma electrolytes, because we confirm previous results (Rutherford et al., 2005) that such mechanical hyperventilation has negligible effects on electrolyte levels. The only significant electrolyte difference we found was in Ca^2+^ levels (a significant fall in hypocapnia of only 0.03 mM). This fall is so trivial (ionized Ca^2+^ levels still remained within the normal range of 1.1–1.4 mM) that it is too small to affect T wave height. Moreover we found previously (Rutherford et al., 2005) that hypocapnia produced a bigger T wave effect with no significant effect on plasma Ca^2+^ levels (1.23 ± 0.01 mM). In any event, it is K^+^, rather than Ca^2+^ flux, that is primarily associated with the repolarization phase of the cardiac action potential.

We also confirm that hypocapnia induced by mechanical hyperventilation produces negligible changes in heart rate and blood pressure in healthy subjects (Cooper et al., 2003, 2004; Rutherford et al., 2005; Parkes et al., 2014) and in patients with breast cancer (Parkes et al., 2016b).

The methodological advantage of demonstrating that mechanically induced hypocapnia does consistently produce significant effects even in healthy subjects is that this offers an in-built positive result. So the only further requirement is that this positive effect is bigger in patients with coronary artery disease.

### Hypocapnia in Angina Patients

Our patients had typical and debilitating angina with angiographically confirmed severe epicardial coronary artery disease (warranting surgical intervention). Hypocapnia to a mean PetCO_2_ of 20 ± 0 mmHg did produce statistically significant changes in the T wave of the ECG in our angina patients. But in none did it cause angina. It is possible that these ECG changes reflect some hypocapnia-induced coronary artery constriction. Further angiography during mechanical hyperventilation would confirm this. Simultaneous comparison with the results of stress echocardiography, myocardial-scintigraphy and stress cardiac MRI in a larger number of patients would provide definitive comparison of all such techniques. Nevertheless these effects of hypocapnia on the ECG are no greater than those in healthy subjects and are well below the AHA clinical threshold. So a means of enhancing any coronary vasoconstricting effects of hypocapnia is an obvious next step.

There are a few case reports of single patients being admitted because of spontaneous hyperventilation to 15 mmHg (Kruyswijk et al., 1986) or 12 mmHg (Freeman and Nixon, 1985) and experiencing angina, but such PaCO_2_ levels would be considered unsafe to impose on patients for a routine clinical test. So we do have a positive result that confirms hypocapnia at 20 mmHg still has effects in angina patients. But without producing bigger effects on the ECG than in healthy subjects, or causing angina, hypocapnia alone is not yet a clinically useful tool. Why hypocapnia has no greater effects in angina patients is unclear. Possibly hypocapnia only constricts the resistance vessel less distal to the stenosis (because of endogenous production of vasodilators), which therefore reduces the pro-ischemic effect. Or possibly the stenosis itself subsequently renders the surrounding coronary artery tissue insensitive to hypocapnia.

### Strengths and Limitations

We did not recruit female patients, although we have no evidence that females might be more responsive to hypocapnia. Similarly, although our healthy subjects are not age matched with our patients, this is of little importance. Each subject is their own control and the older group is if anything less, rather than more responsive to hypocapnia, so our data is not biased toward a positive result.

We showed previously in healthy volunteers (Rutherford et al., 2005) that mechanical hyperventilation is superior to voluntary hyperventilation in inducing longer and stable periods of more severe hypocapnia. Mechanical hyperventilation in conscious and unsedated patients with angina was simple, safe and straightforward. All 10 patients tolerated it well with minimal discomfort and all returned on multiple occasions to complete the study. Some were ventilated for up to 1 h and none objected to this. Experiments, however, usually lasted ∼30 min, but could be further shortened if required. Our demonstration with angina patients of the safety and acceptability of mechanical hyperventilation and hypocapnia is an important milestone in the advocacy of non-invasive mechanical hyperventilation by facemask.

### Implications for the Search for Non-invasive Tests of Early Coronary Artery Disease

It is always encouraging for new and non-invasive procedures where mild hypocapnia (∼30 mmHg), induced by voluntary hyperventilation, produces myocardial changes detectable in healthy subjects. But voluntary hyperventilation produces notoriously variable results in cardiology due the variability in achieved inflation volumes, duration and stability of hypocapnia and requires considerable co-operation of the subject (Rutherford et al., 2005).

Our current and previous results show that even well conducted studies only on healthy volunteers are not in themselves clinical validation that such procedures will have any clinical diagnostic value. We have not tested any of the more recently described new and non-invasive cardiac diagnostic techniques ourselves, nor made direct comparisons with known pharmacological diagnostic procedures. But the overriding result from our angina patients is that severe and prolonged hypocapnia alone never itself produced angina. This is even when applying the more rigorous mechanical hyperventilation to induce more severe and prolonged hypocapnia (∼20 mmHg). We therefore establish that even this degree of hypocapnia does not at present reach the benchmark that such tests should consistently produce angina and greater ECG and other cardiac changes in patients with coronary artery disease. Clinicians may therefore be circumspect in considering any claims for hypocapnia, on its own, being worth further clinical evaluation without the further modifications that we now propose below.

There is still a need for low cost, practical and sensitive diagnostic tests for early coronary artery disease that are non-invasive and avoid pharmacological interventions (e.g., adenosine). Mechanically induced hypocapnia still has potential because it is so simple, safe, comfortable and easy to apply. One possible new means of enhancing any coronary vasoconstricting effects of hypocapnia would be to combine it with a lesser degree of hypoxia than used in previously described studies. (This would be instead of the more usually considered asphyxia i.e., hypoxia with hypercapnia). Early studies using inspiration of hypoxic gas mixtures (8–12% oxygen) in angina patients did consistently produce pronounced ST and T wave ECG changes (Levy et al., 1939, 1941; Turner and Morton, 1952; Haarstad and Broch, 1958; Broch, 1972) and angina (Levy et al., 1939, 1941; Turner and Morton, 1952; Haarstad and Broch, 1958). Initially this was considered as a possible diagnostic test for early coronary artery disease. Its sensitivity and specificity was believed as good or better than exercise testing (Levy et al., 1941; Turner and Morton, 1952; Stewart and Carr, 1954; Haarstad and Broch, 1958). But severe hypoxia was abandoned in the 1950s because hypoxia did induce angina (Levy et al., 1939, 1941; Turner and Morton, 1952; Haarstad and Broch, 1958), patient distress, and had the potential for adverse risks including myocardial infarction or death (Stewart and Carr, 1954).

More modern and non-invasive oxygen and heart monitoring combined with better patient screening may, however, make such a hypoxia challenge much safer. So, the next approach could be first to test whether combining hypocapnia with a milder level of hypoxia that is clinically safe, might produce greater and easily measurable effects in patients with ischemic heart disease. If so, this combined hypocapnia-with-hypoxia test could then be compared for efficacy, safety, and ease of use with the range of tests for coronary artery disease currently in use. It could then be tested also how well such positive results predict angina that develops at a later stage and therefore detects for the first time clinically silent coronary artery disease.

## Conclusion

In healthy subjects, hypocapnia has long been known to cause severe constriction of epicardial resistance vessels, reductions in coronary artery blood flow, myocardial oxygenation and small changes in the T wave of the ECG. While it is increasingly believed that hypocapnia might offer some clinical benefit as a non-invasive diagnostic tool for early coronary artery disease, nobody has previously tested whether its effects are any greater in patients with angiographically established coronary artery disease.

We show that non-invasive mechanical hyperventilation in awake and unmedicated patients with angina is acceptable and safe. We applied more severe hypocapnia and for longer periods than previously, but we show that the effects of hypocapnia alone are no greater in patients with angiographically established coronary artery disease. The goal of developing a simple, safe and non-invasive test of early coronary artery disease is still being pursued. What may be done next is to test whether larger and clinically important effects in patients with ischemic heart disease occur if such severe hypocapnia is combined with mild hypoxia.

## Data Availability Statement

The datasets for this study will not be made publicly available because of patient ethical restrictions.

## Ethics Statement

Experiments were conducted in the NIHR/WTCRF at the University Hospital Birmingham. All participants gave informed consent and all experiments were approved by the Walsall Local Research Ethics Committee and were performed in accordance with the Declaration of Helsinki and Good Clinical Practice.

## Author Contributions

MP, TC-B, and MF designed and applied to fund this study. MP, JS, TB, AR, and ES collected the data. MP and JS analyzed the data. All authors interpreted the data, wrote the manuscript, and approved the final draft.

## Conflict of Interest

The authors declare that the research was conducted in the absence of any commercial or financial relationships that could be construed as a potential conflict of interest.
